# Reversible cell cycle inhibition and premature aging features imposed by conditional expression of p16^Ink4a^

**DOI:** 10.1111/acel.12279

**Published:** 2014-12-06

**Authors:** Amelie Boquoi, Sanjeevani Arora, Tina Chen, Sam Litwin, James Koh, Greg H Enders

**Affiliations:** 1 Cancer Biology Program, Fox Chase Cancer CenterPhiladelphia, PA, USA; 2 Department of Medicine, Fox Chase Cancer CenterPhiladelphia, PA, USA; 3 Department of Biostatistics, Fox Chase Cancer CenterPhiladelphia, PA, USA; 4 Department of Surgery, Duke University Medical CenterDurham, NC, USA

**Keywords:** aging, Cdk, Ink4a, p16, senescence, stem cell

## Abstract

The cyclin-dependent kinase (Cdk) inhibitor p16^Ink4a^ (p16) is a canonical mediator of cellular senescence and accumulates in aging tissues, where it constrains proliferation of some progenitor cells. However, whether p16 induction in tissues is sufficient to inhibit cell proliferation, mediate senescence*,* and/or impose aging features has remained unclear. To address these issues, we generated transgenic mice that permit conditional p16 expression. Broad induction at weaning inhibited proliferation of intestinal transit-amplifying and Lgr5+ stem cells and rapidly imposed features of aging, including hair loss, skin wrinkling, reduced body weight and subcutaneous fat, an increased myeloid fraction in peripheral blood, poor dentition, and cataracts. Aging features were observed with multiple combinations of p16 transgenes and transactivators and were largely abrogated by a germline Cdk4 R24C mutation, confirming that they reflect Cdk inhibition. Senescence markers were not found, and de-induction of p16, even after weeks of sustained expression, allowed rapid recovery of intestinal cell proliferation and reversal of aging features in most mice. These results suggest that p16-mediated inhibition of Cdk activity is sufficient to inhibit cell proliferation and impose aging features in somatic tissues of mammals and that at least some of these aging features are reversible.

## Introduction

Aging in mammals is a complex process that is characterized by accumulation of macromolecular damage and compromised tissue renewal, but the regulation of renewal and its impact on aging remain incompletely defined. Most current mouse models of aging are underpinned by DNA damage (Hasty & Vijg, 2004). The tumor suppressor and cell cycle inhibitor p16^Ink4a^ (p16) accumulates in aging tissues of mice and humans and has been proposed as a biomarker of aging (Zindy *et al*., 1997; Janzen *et al*., 2006; Krishnamurthy *et al*., 2006; Molofsky *et al*., 2006; Liu *et al*., 2009). Further, its role in mediating cellular senescence in settings of extended cell proliferation *in vitro* (Alcorta *et al*., 1996), oncogene activation (Serrano *et al*., 1997), and DNA damage (Schmitt *et al*., 2002) adds to its appeal as a potential effector of aging.

The p16 response pathway has been well defined. p16 binds to cyclin-dependent kinases (Cdks) 4 and 6, displacing D cyclins (Sharpless & DePinho, 1999). Cdk2 can also be potently inhibited, primarily through redistribution of p21^CIP1/KIP1^ class proteins from Cdk4/6 complexes, rendering p16 a robust interphase Cdk (iCdk) inhibitor (McConnell *et al*., 1999; Mitra *et al*., 1999). Phosphorylation of retinoblastoma (pRb) family proteins is thereby inhibited, stabilizing their binding to E2F transcription factors and repressing many genes involved in cell replication. Point mutations in p16 found in tumors regularly abrogate its ability to bind to Cdk4/6 and inhibit cell cycle entry (Koh *et al*., 1995). Familial melanoma can be caused by mutations in p16 or Cdk4. The melanoma-associated Cdk4 R24C mutation disrupts p16 binding (Rane *et al*., 2002). Expression of p16 in pRb-null cells has little or no discernible effect (Koh *et al*., 1995; Enders *et al*., 1996). Thus, p16 constitutes a well-defined inhibitor of iCdk activity and proliferation of tumors cells and many normal cells in culture.

However, the role of this pathway in regulating proliferation of normal adult cells in mammals has been seriously challenged. Individual null mutations (‘knockouts’) of iCdks in mice have yielded subtle and/or highly tissue-specific phenotypes (Satyanarayana & Kaldis, 2009). Combined knockouts of Cdk2 and 4 or Cdk4 and 6 cause late embryonic lethality, associated with cardiac defects and anemia, respectively. Most cell proliferation appears normal, however, as in early embryos devoid of iCdks (Santamaria *et al*., 2007). Similarly, knockouts of iCdk cyclins yielded highly tissue-specific deficits (Kozar *et al*., 2004). Finally, proliferation of the small intestinal epithelium, the most highly proliferative tissue in adults, was not impaired in mature mice rendered conditionally null for all three activating E2Fs (Chong *et al*., 2009).

Levels of p16 increase with age in several tissues *in vivo* (Zindy *et al*., 1997; Nielsen *et al*., 1999; Krishnamurthy *et al*., 2004). Moreover, aging p16-null mice display greater preservation of proliferation of progenitor cells in pancreatic islets, bone marrow, and some neural tissues (Janzen *et al*., 2006; Krishnamurthy *et al*., 2006; Molofsky *et al*., 2006). Based on such observations, p16 has been proposed as a biomarker and potential effector of aging and is widely used as a marker of cellular senescence, *in vitro* and *in vivo*. Nonetheless, the effect of p16 on aging *per se* is difficult to assess in p16-null mice, because they are tumor-prone (Sharpless *et al*., 2004).

To directly test the impact of p16 expression on cell proliferation, senescence, and aging features in adult tissues, we generated transgenic mice in which p16 can be conditionally expressed. We found that p16 induction strongly inhibited proliferation of normal intestinal cells of young adult mice and rapidly imposed several features of premature aging. De-induction of p16 revealed that these features were strikingly reversible.

## Results

### Generation of transgenic mice with inducible p16 expression

We chose to induce human p16 because it is indistinguishable from mouse p16 in ability to arrest mouse cells *in vitro* (Koh *et al*., 1995; Quelle *et al*., 1995; Enders *et al*., 1996) and can be sensitively detected with the monoclonal antibody JC2 (Nielsen *et al*., 1999; Dai *et al*., 2000). This antibody does not efficiently recognize mouse p16 (see below). We used a well-characterized construct in which the p16 cDNA is cloned behind a tetracycline (‘Tet’) operon (Fig. S1, Supporting information). This construct permits robust Tet-regulated p16 expression in cultured cells (Mitra *et al*., 1999; Dai & Enders, 2000). We generated three independent founder lines (‘TetO-p16’). PCR amplification and DNA sequencing showed the transgene coding regions to be intact. TetO-p16 mice were generally healthy and fertile. A few developed bilaterally symmetric patches of alopecia around 1 month associated with some ‘leaky’ p16 expression, but this phenotype disappeared during backcrossing into a Bl6 background (< 1/30 mice of each TetO-p16 line).

### p16 induction

**Figure 1:**
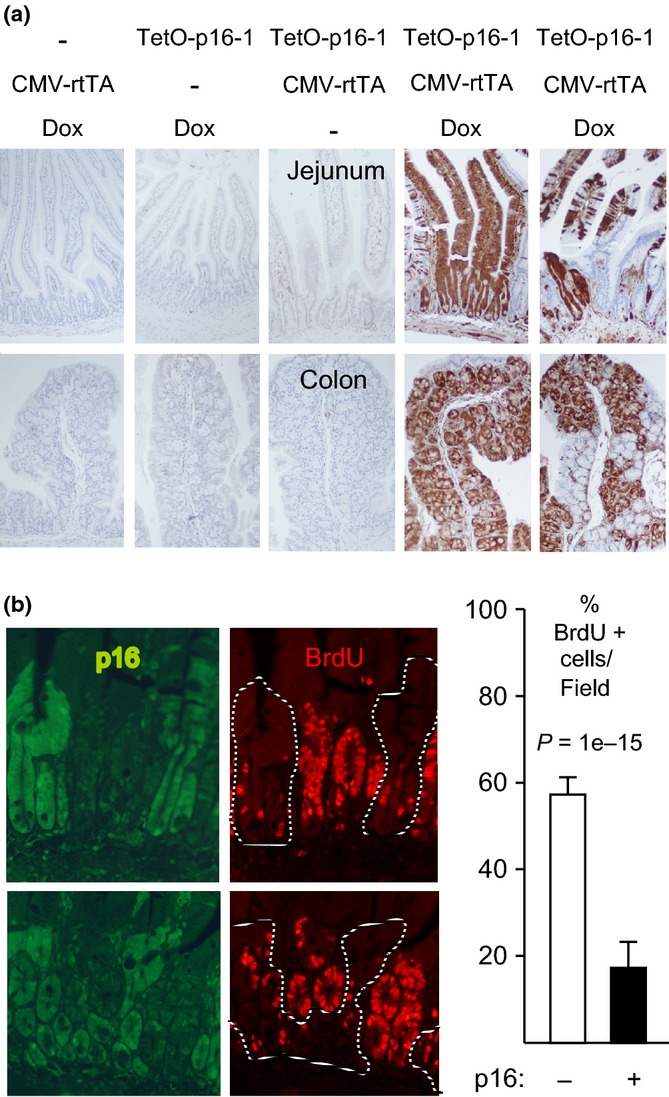
p16 induction inhibits intestinal epithelial cell proliferation. (a) Mice of different genotypes were or were not treated with Dox for 1 week, as designated. IHC for exogenous p16 (brown, 10× fields). Note strong mosaic p16 induction in the presence of both transgenes and Dox. (b) CMV-rtTA:TetO-p16-1 mice treated with Dox for 1 week and injected with BrdU. Panels (left): Two 20× fields (above, below) with co-IF for p16 (left, green; right, dashed lines) and BrdU (right, red). Graph (right): Among 1122 crypt cells scored, 19% of p16+ cells were BrdU+ vs. 58% of p16- cells. Modest BrdU signal was visible in the green filter.

To examine regulation of the transgene, we mated TetO-p16 mice to mice that express the reverse Tet transactivator (‘Tet-on’) from a human cytomegalovirus promoter (‘CMV-rtTA’ [Sotillo *et al*., 2007]). The vigorous proliferation of the intestinal epithelium makes it an attractive setting for studies of tissue renewal. Further, p16 suppresses tumorigenesis in the intestine (Gibson *et al*., 2005; Furth *et al*., 2006). Treatment of 3-month-old CMV-rtTA:TetO-p16 mice with doxycycline (‘Dox’) for 1 week broadly and strongly induced p16 in intestine and other tissues examined with little or no detectable ‘leaky’ expression without all three factors (Fig.1a, Fig. S2, Supporting information). p16 induction patterns and levels were similar in all TetOp-16 lines. We used them interchangeably, with indistinguishable results. Expression peaked by day 6 (d6) and was mosaic (Fig1a), consistent with previous experience with the CMV-rtTA line (K. Politi and H. Varmus, personal communication). Immunoblotting (IB) and immunoprecipitation (IP) from isolated intestinal epithelial cells (Weiser, 1973) showed that induced p16 was expressed at levels moderately higher in colon than in jejunum or our p16-inducible U2-OS cells (Mitra *et al*., 1999), bound to Cdk4, and was associated with reduced expression and phosphorylation of pRb (Fig. S3a–c, Supporting information). In K5-rtTA:TetO-p16 mice (Missero *et al*., 1993), selective expression was observed in squamous epithelium (Fig. S4, Supporting information), illustrating the tight control and versatility of the TetO-p16 transgene.

### p16 inhibits cell cycle progression *in vivo*

To assess whether p16 induction inhibited cell cycle progression, we treated young (3 month old) CMV-rtTA:TetO-p16 mice with Dox for 1 week. Intestinal tissue was costained by immunofluorescence (IF) for p16 and bromodeoxyuridine (BrdU). The mosaic expression pattern permitted comparison of p16-expressing to nonexpressing cells in the same tissue. p16+ cells displayed 65% less BrdU incorporation than neighboring p16- cells (Fig.1b; *P* = 1e-15; TetO-p16-1 line). p16 expression also reduced staining for PCNA, another marker of S phase (Fig. S5a, Supporting information, *P* = 0.007; TetO-p16-2 line), and phospho-histone H3, a marker of mitosis (P-H3, Fig. S5b, Supporting information, *P* = 0.003; TetOp16-2 line). We conclude that p16 induction is sufficient to inhibit proliferation of intestinal epithelial cells.

### p16 inhibits intestinal stem cell proliferation

**Figure 2:**
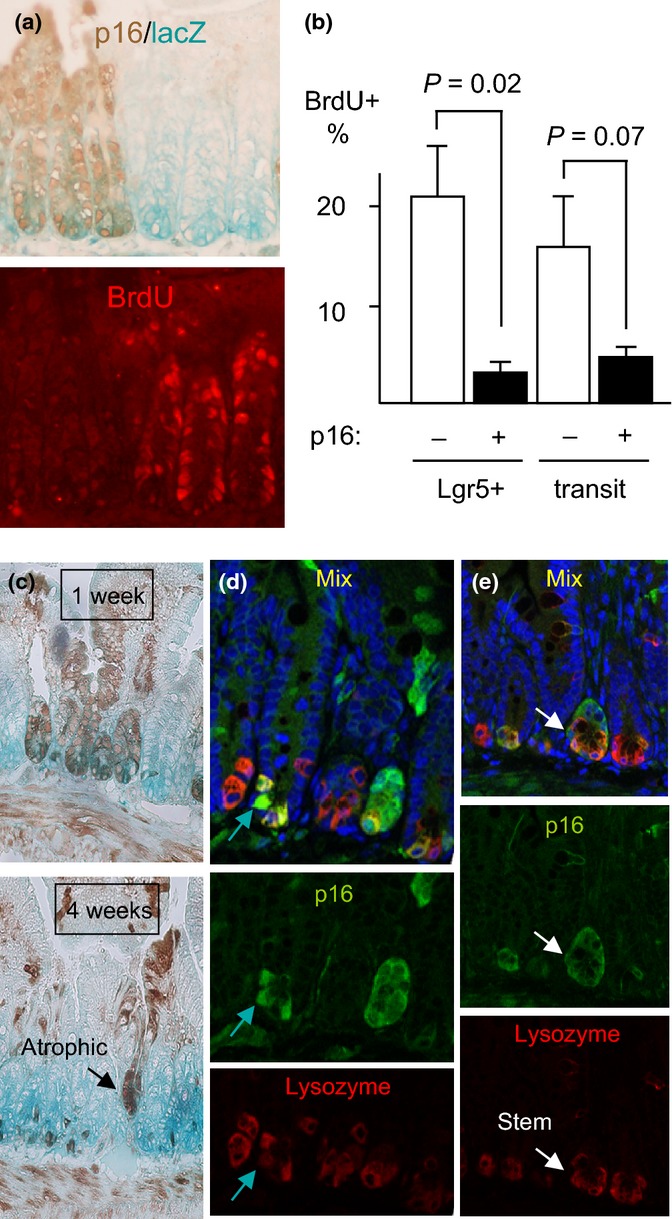
Induced p16 inhibits proliferation of intestinal stem cells. (a) CMV-rtTA:TetO-p16:Lgr5-lacZ mice were treated with Dox for 1 week and injected with BrdU. LacZ was detected by activity stain (left, aqua), p16 by IHC (left, brown), and BrdU in the same section by IF (right, red). Note paucity of BrdU staining in the p16+ Lgr5-lacZ+ cells (left 3 crypts). Across 8 20× fields, p16+ Lgr5+ cells (*N* = 331) showed no BrdU staining vs. 15% of the p16- Lgr5+ cells (*N* = 461, *P* = 0.02) (b) In the same mice, p16 and BrdU were detected by IHC in serial 4- to 5-micrometer sections and the % BrdU+ cells were scored in Lgr5+ and transit-amplifying cells, respectively. P values by two-sided *t*-tests. The one-sided *t*-test for transit cells = 0.03. *N* = 3 mice; 5803 cells counted. (c) Reduced p16 expression in small intestines of mice with continuous p16 induction. CMV-rtTA:TetO-p16:Lgr5-lacZ mice were treated with Dox near 2 mos of age for 1 or 4 weeks. IHC for p16 (brown) and activity assay for lacZ (aqua). Fields 10x. *N* = 3 mice per time point. Note the gradual loss of p16 expression, with preferential retention at the crypt base (*P* = 0.02 vs. transit-amplifying zones, *P* = 0.007 vs. villi). An atrophic, p16+ crypt is marked. (d, e) Partial costaining for p16 and lysozyme in crypt base cells, detected by confocal co-IF. Mice treated with Dox d20-40 were stained for p16 (green), lysozyme (red), and DNA (DAPI, blue; top). Green arrows (d) mark a p16+ cell that stains weakly for lysozyme, reflecting partial paneth cell differentiation. White arrows (e) identify a p16+ lysozyme- cell that is surrounded by lysozyme+ cells and, hence, is likely a stem cell.

Intestinal stem cells support renewal of the epithelium. Lgr5+ stem cells are confined to the crypt base, where they are the only major proliferative cell (Barker *et al*., 2007). Therefore, crypt base proliferation is a good measure of Lgr5+ stem cell proliferation. To further identify these cells, we mated CMV-rtTA:TetO-p16 mice to Lgr5-lacZ mice (Barker *et al*., 2007). Intestinal sections were costained for p16, BrdU, and lacZ activity. p16+ crypt base cells in the Lgr5-lacZ+ zone showed > 90% lower BrdU staining than nearby p16- Lgr5-lacZ+ cells (Fig.2a, *P* = 0.02). Some diffusion of the lacZ stain occurred during IHC, but it helped confirm the stem cell zone and was not reduced by p16 induction. To avoid potential masking of BrdU staining by p16 staining, we confirmed this observation using serial stained sections. In such sections, p16+ Lgr5+ cells displayed 85% lower BrdU staining than neighboring p16- Lgr5+ cells (Fig. S5c, Supporting information).

When p16 was induced for 4 weeks, expression was preferentially reduced in transit-amplifying zones and villi but retained in scattered crypt base cells (Fig.2c, *P* = 0.02). Residual crypts that were strongly p16+ appeared atrophic. These observations were consistent with arrest of p16+ stem cells and replacement of much of the epithelium by p16- cells. p16 induction did not spontaneously diminish in the proliferative compartment: In mice sacrificed at similar ages after only 1 week of p16 induction, 45% of transit-amplifying zones showed p16 staining vs. 16% in mice with continuous induction for 60 days (four mice per group; 651 crypts, 643 transit-amplifying zones, 230 villi scored; *P* = 0.02). Similarly, replacement of ATR-null intestinal epithelial cells by ATR-expressing cells has been described following acute ATR gene deletion, albeit in a model that involves DNA damage (Ruzankina *et al*., 2007). Staining for activated caspase 3, an apoptosis marker, showed no detectable increase in p16+ tissue (Fig. S6, Supporting information). p16-expressing cells may be lost by other means, such as sloughing. We conclude that p16 induction in intestinal stem cells inhibits their production of transit-amplifying and villous cells.

To further clarify the cell types that expressed p16 after sustained induction, we performed co-IF with markers of differentiation. Following induction for 1 week, p16 staining was seen in all cell types, without bias. After 4 weeks of induction, fewer crypts and overlying villous tracts exhibited uniform p16 staining (Fig.2c,d; Fig. S7a, Supporting information). The majority of p16+ crypt base cells costained for lysozyme, a sensitive marker of paneth cell differentiation (Fig.2d, Fig. S7, Supporting information blue and yellow arrows). Paneth cells are differentiated, nonmigratory, long-lived cells. Arrest of intestinal crypt base cells mediated by conditional loss of Cdc25 cell cycle activators was associated with partial paneth cell differentiation within 1 week (Lee *et al*., 2009). Although no major increase in paneth cell number was seen following p16 induction, some p16+ stem cells may adopt this fate (Fig. S7e, Supporting information). Some p16+ crypt base cells 8 weeks after induction were lysozyme- and, therefore, appear to be stem cells (Fig.2e, Fig. S7d, Supporting information; white arrows). The absence of p16+ transit-amplifying cells above them confirmed their lack of proliferation.

p16+ cells generally also lacked markers of goblet cells (mucin vacules and mucicarmine staining) (Fig.2c–e, Fig. S7a–d, Supporting information). Strong p16 induction for 3–6 days in several established human cell lines is sufficient to impose senescence in most cells, with associated ß-galactosidase activity (‘SAß-gal’) and irreversible loss of proliferative potential (Dai & Enders, 2000; Beausejour *et al*., 2003). Induction for 1 or 3 weeks did not detectably increase SAß-gal staining in intestine (Fig. S8, Supporting information).

These observations suggest that p16 expression was sufficient to inhibit cell cycle progression in Lgr5+ intestinal stem cells. Over weeks, crypts with uniform p16 expression may atrophy and be replaced, whereas isolated stem cells with p16 expression appeared to remain arrested. Over time, some such cells may develop paneth cell differentiation and/or slough, but SAβgal staining was not detected.

### p16 induction imposes premature aging features

We examined whether p16 induction in young mice could impose premature aging features. CMV-rtTA:TetO-p16 mice and singly transgenic and wild-type littermate controls were treated with Dox chow at weaning, postnatal d20. Dox was continued to d40, chosen to span the first wave of hair regeneration. No phenotypic differences were observed between singly transgenic and wild-type mice, so their results were combined in the analyses. Bitransgenic mice showed reduced hair density over the entire coat, variable lightening of hair color, lower weight, and kyphosis (Fig. S9b, d, f, Supporting information) compared to controls (Fig. S9a, c, e, Supporting information; Table1). Hair density on the back of treated bitransgenic mice was 25% of control levels (Table1, *P* = 0.0001). We used this potent effect to document quantitatively similar phenotypes in the two TetO-p16 strains used (line 1: 27%, *P* = 0.0009; line 2: 24%, *P* = 0.002), confirming its independence from mutations that might have occurred during TetO-p16 transgene integration. The hair was also thinner in diameter (Table1, *P* = 0.03). Histological analysis revealed strong mosaic p16 induction in skin, including Lgr5-lacZ+ hair follicle stem cells (Fig. S10a-d, Supporting information). A mix of short and long hair follicles were seen, indicative of a disordered anagen. These results demonstrate that p16 induction inhibited hair regeneration.

**Table 1:** Aging features quantified following p16 induction in CMV-rtTA mice d20-40†

	Littermate controls†††		Bitransgenics			
	*N*	Mean ± SD	*N*	Mean ± SD	%‡‡‡	Significance§§§
Hair density‡	14	12.3 ± 1.7	9	3.1 ± 0.8	25	*P* = 0.0001
Hair diameter§	3	1.27 ± 0.16	3	0.75 ± 0.05	59	*P* = 0.03
Body weight¶	17	19.6 ± 2.5	8	17.3 ± 2.9	89	*P* = 0.02
SI length††	17	26.9 ± 3.3	8	21.9 ± 3.6	81	*P* = 0.002
Colon length‡‡	17	6.0 ± 0.6	8	4.9 ± 0.7	82	*P* = 0.0006
Hematocrit§§	13	57 ± 8.4	8	53 ± 4.3	92	*P* = 0.18
Myeloid fraction¶¶	9	6.0 ± 6.8	6	12.8 ± 7.3	210	*P* = 0.02

† All scoring was blinded to the mouse genotypes.

‡ mg/cm^2^ shaved from mid-back.

§ Arbitrary units, hair shaved from mid-back; a total of 309 hairs scored

¶ g, males and females combined; males alone also significantly different.

†† cm

‡‡ cm

§§ % Packed blood cell volume.

¶¶ % Myeloid cells among white blood cells in blood smears.

††† Wt and single transgene results did not differ significantly and were pooled.

‡‡‡ Bitransgenic value as % of control.

§§§ By Wilcoxon tests.

There was a paucity of subcutaneous and abdominal fat in bitransgenic mice treated d20-40. Body weight was 11% lower (Table1, *P* = 0.02). Small intestine and colon were 18–19% shorter (Table1; *P* = 0.002 and *P* = 0.0006, respectively). Similar intestinal shortening was seen previously in mice with conditional loss of Cdc25 activity (Lee *et al*., 2009). Although intestinal length has not been measured in aging humans and mice, they show reduced absorption of nutrients. Aging humans and mice also manifest a mild anemia with a shift from lymphoid to myeloid lineages (Janzen *et al*., 2006). There was a trend toward mild anemia in bitransgenic mice (Table1). Blood smears revealed a myeloid shift (Table1, *P* = 0.02). In summary, these observations suggest that p16 induction at weaning is sufficient to impose features of aging in multiple tissues by 6 weeks of age, among the most rapid premature aging models described (Hasty & Vijg, 2004; Kirkwood, 2005).

### Reversibility of aging features in CMV-rtTA mice

**Figure 3:**
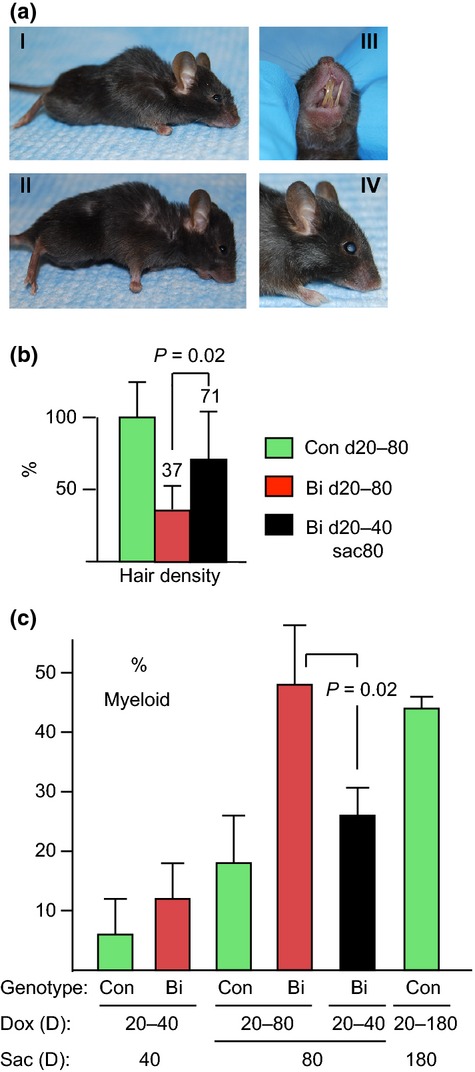
Progression vs. recovery of aging features. CMV-rtTA:TetO-p16 littermates treated with Dox d20-40 were divided into 2 groups. Dox was sustained in one and withdrawn in the other. Mice were sacrificed at d80. (a) Sustained Dox treatment d20-80 results in persistent defects. Note the thin hair, hunched posture, thin body, and sunken eyes in representative mice (a1, all); malocclusion of the incisors, with resulting overgrowth (alll, 5/17 mice); and cataracts (alV, bilateral, confirmed by histology; 2/17 mice). (b) Back hair density (mg cm^−2^) in bitransgenic mice (Bi) treated with Dox d20-80 (*N* = 14) and Bi mice treated with Dox d20-40 and sacrificed at d80 (*N* = 6) were normalized to that of their wild-type and singly transgenic littermate controls (Con) treated with Dox d20-80 (*N* = 12) and depicted as mean ± SD (percentages relative to controls listed). Hair density of Bi d20-40sac80 mice was significantly greater than in Bi d20-80 mice (*P* = 0.02) and did not differ significantly from control mice. (c) Increase in myeloid to lymphoid ratio with sustained Dox treatment d20-80 in bitransgenic mice (*P* = 0.01 compared to control d20-80 mice), ameliorated by discontinuing Dox at d40 (*P* = 0.02 compared to Bi d20-80 mice). Results shown are means ± SD from 3 to 5 mice, except the control d20-180 results, which are mean ± range from two mice.

We then asked whether the effects imposed by p16 were reversible. p16 expression in intestinal epithelial cells from CMV-rtTA:TetO-p16 mice treated for 1 week with Dox was lost within 1 week of Dox withdrawal (Fig. S3d, Supporting information), consistent with data from U2-OS cells (Dai & Enders, 2000). We treated mice with Dox d20-40 and divided them into two groups. Dox was continued in the first group and withdrawn in the second, and mice were euthanized at d80. Sustained Dox treatment in bitransgenic mice was associated with persistently reduced hair density (Fig.3al-ll,b) and a further increase in the myeloid fraction, to a level comparable to that of 180-day-old control mice (Fig.3c). Poor dentition, a new phenotype that may be aging-related, appeared in bitransgenic mice with Dox treatment d20-80 (*N* = 15) or d20-160 (*N* = 2), marked by instability of the incisors with resulting malocclusion and overgrowth (Fig.3alll, 5/17 mice, *P* = 0.05). Two bitransgenic mice developed cataracts (Fig.3alV), although this finding did not reach statistical significance. Two bitransgenic mice reached our criteria for euthanasia by d80, based on reduced spontaneous movements, persistent hunched posture, and decreased grooming, and two died before they could be analyzed at d80. Thus, a total of 4 of 18 bitransgenic mice with sustained induction became debilitated or died by d80 vs. 0 of 12 control mice.

In contrast, when Dox was withdrawn at d40, hair growth rebounded to levels twice that in d20-40 mice (Table1) or d20-80 bitransgenic mice (Fig.3b, *P* = 0.02). To confirm that bitransgenic mice with p16 induction d20-40 (documented in tail snips) could regrow hair, we shaved back hair from such mice at d40. Most mice regrew nearly normal appearing hair by d80 (Fig. S11, Supporting information). The myeloid ratio in bitransgenic mice treated with Dox d20-40 improved by d80 to levels not significantly different than control mice at that age (Fig.3d). Small intestine and colon lengths remained shorter, however (*P* = 0.004 and 0.006, respectively).

### Confirmation of aging features using the M2-rtTA line

Integration of the transgene in the CMV-rtTA line was accompanied by deletion of several genes (R. Benezra, personal communication). To rule out the possibility that the aging effects depended on this heterozygous mutation, we repeated the experiments using a ROSA-M2-rtTA (‘M2-rtTA’) transactivator line. p16 induction in this line was more uniform in epithelial cells of intestine and skin than in CMV-rtTA:TetO-p16 mice, but lower in crypt base cells (Fig. S12a–c, Supporting information). Dox treatment in M2-rtTA:TetO-p16 mice yielded weaker phenotypes than in CMV-rtTA:TetO-p16 mice. Nonetheless, treatment d20-110 caused marked hair loss (Fig. S12d, e, Supporting information; *P* = 0.001), loss of subcutaneous fat, skin wrinkling, lower body weights (*P* = 0.007) and trends toward lower hematocrits and shorter intestines. The more uniform expression of p16 in intestinal epithelium offered an improved opportunity to examine potential biochemical changes in such cells. Nonetheless, real-time reverse-transcription–PCR for senescence marker mRNAs Dec1 and DcR2 (Collado *et al*., 2005) following 1 week of Dox treatment showed no substantial induction (Table S1, Supporting information). In summary, the premature aging phenotypes imposed by p16 induction are independent of transgene integration events.

### Most uniform p16 induction in CAG-rtTA3 mice

During this work, the rtTA transactivator line CAG-rtTA3 was developed and reported to provide the most uniform induction (Premsrirut *et al*., 2011). We therefore bred CAG-rtTA3 mice to TetO-p16 mice. p16 was indeed more uniformly expressed in the intestinal epithelium (Fig. S13a–d, Supporting information), skin (Fig. S10e, Supporting information), as well as other organs (Fig. S14, Supporting information). Use of the CAG-rtTA3 line allowed us to judge effects on cell proliferation in whole tissues. p16 induction for 1 week or 3 weeks (d20-40) reduced overall intestinal epithelial cell proliferation to 52% of uninduced mice (Fig. S15a, Supporting information). Although not as strong as in the subset of p16-expressing intestinal cells in CMV-rtTA mice, this effect represented a greater inhibition of proliferation of the intestinal epithelium as a whole and came despite an apparent feedback loop that acts to sustain proliferation of the epithelium (see below).

**Figure 4:**
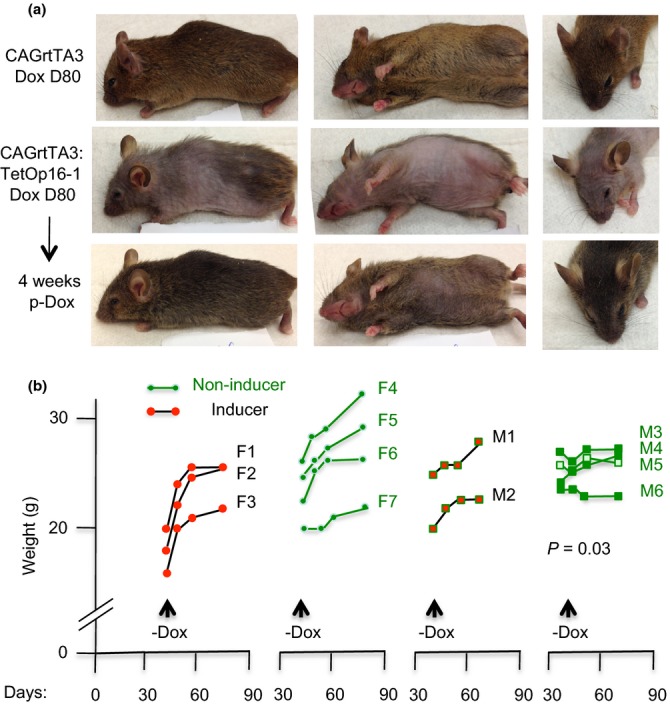
Rapid reversal of aging features in CAG-rtTA3 mice following p16 de-induction. (a) Top row: a noninducing littermate mouse at D80 of Dox treatment (different angles). Middle row: a CAG-rtTA3:TetOp16 mouse at D80 of Dox treatment. Lower row: the same CAG-rtTA3:TetOp16 mouse following 4 weeks of Dox withdrawal. Note dramatic recovery in body hair and skin wrinkling, filling out of face and eyes, and straightening of whisker hairs. Only the hair on the ventral trunk lagged (center), and the eyes remained sunken. (b) Left: littermate female CAG-rtTA3:TetOp16 mice (‘inducers’ F1-3) and wild-type or singly transgenic mice (‘noninducers’ F4-7) were treated with Dox from D20-40. Mice were weighed weekly for 2 weeks and then 4 weeks after Dox withdrawal (*X*-axis: days). Inducers showed a dramatic rebound in weight (*Y*-axis: g) within the first week. Right: littermate male inducers (M1-2) and noninducers (M3-6) were treated with Dox D20-80, and then Dox was withdrawn. Inducers showed a prompt recovery in weight within 2–4 weeks. The slope of weight gain was greater in the mice with prior p16 induction (*P* = 0.03).

CAG-rtTA3:TetO-p16 mice developed aging features similar to those in CMV-rtTA and M2-rtTA mice, except that the loss of hair regeneration was more pronounced, particularly on the ventral trunk (Fig.4a, middle row). Two CAG-rtTA3 mice treated with Dox for 180d developed cataracts, confirming this feature. Because p16 and cyclin D/Cdk4 complexes are known to regulate proliferation of pancreatic islet cell progenitors (Tsutsui *et al*., 1999; Krishnamurthy *et al*., 2006) and p16 was strongly expressed in islets of CAG-rtTA3 mice (Fig. S14, Supporting information), we tested CAG-rtTA3:TetOp-16 mice treated with Dox d20-40 for diabetes. Blood glucose levels were actually lower in p16-inducing mice (*N* = 5, mean = 139 mg dL^−1^) than noninducing mice (*N* = 5, mean = 212 mg dL^−1^), arguing against diabetes as a cofactor in the aging features. IB of the intestinal mucosa showed no increase in γH2AX following p16 induction d20-40, arguing against DNA damage as a major cofactor (Fig. S15d, Supporting information). Our assays do not exclude moderately increased γH2AX in a minority of cells.

### Rescue of p16 effects in Cdk4R24C mice

To confirm that the effects of p16 induction in this model proceeded through the defined p16 response pathway, rather than a nonspecific effect, we mated CAG-rtTA3:TetO-p16 mice to mice carrying a ‘knockin’ Cdk4 R24C mutation. This germline mutation yields mice that are generally healthy but prone to tumors late in the first year of life (Rane *et al*., 2002). Cdk4 R24C largely abrogated intestinal cell cycle inhibition by p16 induction D20-40, more so when homozygous (Fig. S15a, Supporting information), and rescued aging features imposed by this p16 induction (Fig. S15b,c, Supporting information), confirming that the effects derive from iCdk inhibition.

### p16 effects in CAG-rtTA3 mice are largely reversible

We then used the CAG-rtTA3 line to test further whether p16-imposed cell cycle and aging effects were reversible. Dox was withdrawn in bitransgenic mice following p16 induction d20 to 40. Intestinal BrdU incorporation rebounded strongly 1 week later (Fig. S15a, right, Supporting information). Dox was then withdrawn in mice with well-developed aging features following p16 induction from d20 to 80, and the mice were photographed and weighed weekly. These mice exhibited a rapid and dramatic reversal of aging features (Fig.4a, bottom row), with robust weight gain (Fig.4b; greater than the uninduced mice (*P* = 0.03) and hair growth (Fig.4a). Mice that formerly looked old and debilitated became grossly indistinguishable from their littermates except for reduced hair on the ventral trunk and persistently sunken eyes (Fig.4a, bottom row). These observations indicate that p16 continues to constrain tissue renewal after 2 months of induction but that, once further transcription is blocked, homeostasis in some tissues is promptly restored and some aging features reversed.

Some variability in aging features was seen that was informative. CAG-rtTA3:TetO-p16 mouse M1 in Fig. S16 (Supporting information) is notable for the fact that it maintained weights comparable to its noninducing littermate controls, yet still manifested marked hair loss that was reversible upon Dox withdrawal. This and another such example not shown argue that the p16-imposed block to hair regeneration and the restoration of hair growth upon Dox withdrawal are not due to cachexia and recovery from cachexia.

To begin to address the mechanism(s) responsible for the recovery of tissue homeostasis following Dox withdrawal, we performed microarray analysis of intestinal mucosa with p16 induction d20-40. Cdk4 (2.0×), and cyclin D2 (3.7×, #13 highest named gene) were among the genes with mRNAs higher than in uninduced cells, possibly reflecting feedback regulation that strives to sustain cell proliferation (Fig. S17, Supporting information). IHC confirmed higher level expression of Cdk4 and cyclin D in epithelial crypts of mice with sustained p16 induction, likely contributing to subsequent tissue renewal (Fig. S13e-f, Supporting information). These observations suggest that tissue progenitor cells in mice with p16 induction may have been kept quiescent or slowly replicating, rather than senescent or differentiated, and rebounded upon Dox withdrawal, driven in part by feedback regulation involving Cdk activity. Consistent with this notion, intestinal Lgr5 mRNA levels in the microarray analysis were not reduced by p16 induction for 3 weeks, arguing against a major reduction of proliferative stem cells; lysozyme or SAβ-gal were not increased (Fig. S8, Supporting information); and the microarray analysis showed no induction of secretory proteins that are often induced at the mRNA level in senescence *in vitro* (Coppe *et al*., 2008) (Fig. S17, Supporting information). Nonetheless, it remains possible that some senescent cells in intestine with p16 induction escaped detection and contributed to the aging features. Lineage tracing studies are warranted to further define the fate of p16-expressing cells.

## Discussion

The tissue defects that develop with sustained p16 induction in young TetO-p16 mice suggest that iCdk activity is essential for tissue homeostasis. Even though knockout studies show that no individual iCdk is strictly essential, our data suggest that iCdks maintain cell proliferation and forestall aging features in postnatal somatic tissues, when expressed at normal levels. This observation dovetails well with recent findings using gene replacement *in vitro* with analog-sensitive forms that, when expressed at normal levels, Cdk2 is required for efficient cell cycle entry in human and mouse cells (Merrick *et al*., 2011). The phenotype of mice with p16 induction also matches well that recently reported in mice with induction of the iCdk inhibitor p27 (Pruitt *et al*., 2013). Although p27 has been reported to affect other cell properties, such as migration (Pruitt *et al*., 2013), the combined findings argue strongly that loss of somatic cell proliferation due to iCdk inhibition compromises tissue renewal. TetO-p16 mice offer a new model to selectively manipulate the proliferation of cells *in vivo* and address a variety of issues in mammalian development and aging. Our findings also inform efforts to target Cdk activity in adults for cancer therapy, cancer chemoprevention, or treatment of autoimmune disease (Shapiro, 2006).

Natural aging is a complex process in which macromolecular damage, cell death, and loss of proliferative capacity are intertwined. The effects observed here suggest that inhibition of cell proliferation *per se* is sufficient to account for several archetypal aging features. Endogenous p16 levels are generally lower but do reach those observed here in some cells, during aging and settings of renewal under duress (Dai *et al*., 2000; Furth *et al*., 2006). Given that many p16-expressing cells are outcompeted by their neighbors and may be gradually lost to sloughing, cell death, differentiation, and phagocytosis, etc., examining endogenous expression levels of p16 at any given point may underestimate its cumulative impact. A modest increase in p16, generated by increased gene copy number, has been associated with reduced proliferation of pancreatic progenitor cells in older mice (Krishnamurthy *et al*., 2006). On the other hand, increased copy number of the Ink4a/Arf locus as a whole was associated with longevity in mice (Matheu *et al*., 2009). The latter effect may be accounted for in part by Arf and by p16 expression at levels much lower than in our study. In normal aging, loss of tissue renewal may also reflect the expression of other endogenous Cdk inhibitors (Janzen *et al*., 2006), together with reduced expression of cyclins and Cdks and changes in regulatory Cdk phosphorylation, etc. Irrespective of the role of endogenous p16, our results indicate that iCdks are important drivers of cell proliferation in normal young adults and that loss of their activity can account for some features of aging. Further work, including tests of intestinal function, will be needed to clarify the specific tissues and cell types that may be responsible for the aging features.

Whether natural aging features are reversible is unknown. Our finding that some aging features are reversible provides new insight and is consistent with recent evidence that some aging features caused by telomere dysfunction are partially reversible (Jaskelioff *et al*., 2011). Thus, some age-associated tissue dysfunction due to loss of cell proliferation might be ameliorated, if proliferation can be enhanced. This approach might increase risk of neoplasia but potentially provide clinical benefit. Conversely, our studies suggest that side effects from pharmacological Cdk inhibition for chemoprevention or chemotherapy of neoplasia might be reversible.

Our results suggest that strong p16 expression may not suffice to impose senescence in some normal cells of young adult mice. This observation suggests that some caution is warranted in equating p16 expression with senescence *in vivo*. A minority of mice and some tissues did not recover from p16 induction, possibly due to secondary effects beyond repair. This is consistent with the lack of recovery reported recently in mice following induction of the Cdk inhibitor p27 for 8-12 months (Pruitt *et al*., 2013). Mice with p16 induction between d20-80 exhibited a somewhat slower recovery than those with induction d20-40. If borne out by further experiments, this difference may reflect a cell-autonomous decline in proliferation potential in p16-expressing cells, erosion in noncell-autonomous factors (circulating and/or niche) that foster recovery, or secondary effects of tissue dysfunction. Additional studies are warranted to better define the contexts in which p16 can impose senescence *in vivo*. One can speculate that p16 might preferentially induce senescence in settings of DNA damage and neoplasia.

## Experimental procedures

### Mice

The Institutional Animal Care and Use Committee approved all animal work. TetO-p16 mice were generated by standard pronuclear injection of a p16 transgene derived from pUH10-3, previously described (Mitra *et al*., 1999; Dai & Enders, 2000), and liberated by Xhol and Hindlll. The p16 sequence starts at nucleotide 237 of the NCBI reference standard NM_058197.3. The transgene was identified by PCR primers AGCTCGTTTAGTGAACCGTCA (promoter) and CCTCCGACCGTAACTATTCG (p16). Mice were generated in C57Bl/6 × C3 backgrounds and maintained on standard chow and day/night cycles. CMV-rtTA mice were generated by H. Varmus (National Cancer Institute) and kindly provided in a C57Bl/6 background by R. Benezra (Memorial Sloan Kettering Cancer Center). All CMV-rtTA studies were carried out using the TetO-p16-1 and TetO-p16-2 lines. ROSA M2-rtTA and CAG-rtTA3 transgenic mice were obtained in C57Bl/6 genetic backgrounds from Jackson Laboratories (stock #s 006965, 007678, and 016532, respectively). Lgr5-lacZ transgenic mice were obtained in a 129Sv background from H. Clevers (Vereniging Het Nederlands Kanker Instituut, The Netherlands) (Barker *et al*., 2007) and backcrossed twice onto a C57Bl/6 background. K5-rtTA mice were kindly provided in a BALB background by J. Chernoff (Fox Chase Cancer Center). Cdk4 R24C mice were obtained from S. Rane (NIH, USA). Doxycycline was provided in chow (Harlan Laboratories 625 mg kg^−1^). BrdU was injected into the peritoneum (100 μL of a 10 mg mL^−1^ solution) 4 h before sacrifice. All experiments were performed on littermate mice with the potential to inherit each genotype and any residual mixed genetic backgrounds (most experiments were in a largely Bl6 background) and were scored by investigators blinded to the genotypes.

### Histological and molecular

IHC and IF were performed as described previously (Dai *et al*., 2000), using formalin-fixed and paraffin-embedded jejunal sections and the following antibodies: human p16 (JC2, Jim Koh, Duke University), mouse p16 (M156, Santa Cruz Biotechnology, Santa Cruz, CA, USA) BrdU (IHC: Becton Dickinson, #347580; IF: Abgene), activated Caspase 3 (#9661, Cell Signaling Technology, Danvers, MA, USA), lysozyme (Dako, # EC.3.2.1.17), chromogranin A (Immunostar, #20085), PCNA (#2586, Cell Signaling), phospho-histone H3 (#9071, Cell Signaling), cyclin D1 (sc-753 (cross-reacts with cyclin D2, Santa Cruz), Cdk4 (#sc-260 (Santa Cruz), and γH2AX (#05-636, EMD Millipore, Billerica, MA, USA). Lgr5-lacZ expression was assayed by a modification of methods described previously (Barker *et al*., 2007). Freshly excised tissue was fixed in 2% neutral buffered formalin/0.2% glutaraldehyde/0.01% deoxycholate/0.2% NP40 for 30 min at RT, then incubated in X-gal substrate at RT overnight in the dark before embedding in paraffin. SAβgal staining was at pH 6.0. For p16 IB and Cdk4 IP, intestinal epithelial cells were isolated by treatment of excised tissue with EDTA (Weiser, 1973) and protein extraction with E1a lysis buffer (Harlow *et al*., 1985). For IB of γH2AX, mucosa was scraped off with a razor blade and chromatin extracts sheared by sonication.

### Aging features and time-dependent changes in p16 expression

Diameter of shaved hairs were measured where they crossed the 10× image border. The hematocrit of central venous blood was determined in a heparinized capillary tube after centrifugation 5 min at 10 000 *g*. Standard blood smears were scored for blue cells with round nuclei and little cytoplasm (lymphoid) or multilobed or markedly acentric nuclei and more abundant cytoplasm (myeloid). Small intestine length was measured from the duodenal sweep to the cecum, colon from the cecum to the anal canal. For scoring loss of p16 expression following induction for weeks, at least 40 crypt bases, transit-amplifying zones, and villi, respectively, per mouse were scored as 0 = no p16+ cells, 1 = 0–25%, 2 = 25–50%, 3 = 50–75%, 4 = 75–100. SAßgal staining was performed as described [Dai ref] after fixing tissue in paraformaldehyde.

### Statistical analysis

Statistical comparisons were by Wilcoxon two-sample tests unless specified. Fisher's exact test was used to analyze the distribution of expression of endogenous p16 among Lgr5+, transit-amplifying cells, and villous cells and the unstable teeth phenotype. Chi-square tests were used to compare the fractions of p16+ and p16-cells that were BrdU+. The permutation test was used for analysis of cell cycle markers PCNA and P-H3. A random effects model was used when comparing the fraction of p16+ and p16-negative Lgr5+ cells that were BrdU+ in serial sections. The Jonckheere–Terpstra test was used to assess preferential retention of p16 staining in crypt bases vs. transit-amplifying zones and villi. Hair diameters in p16+ and p16-mice were compared by a two-sided *t*-test.

## Supporting Information

**Fig. S1** Structure of the TetO-p16 transgene. **Fig. S2** Broad p16 expression in CMV-rtTA:TetOp-16 mice. **Fig. S3** p16 induction detected by IB and Cdk4 immunoprecipitation (IP) from isolated intestinal epithelial cells. **Fig. S4** Selective p16 induction in squamous epithelium in K5-rtTA:TetOp16 mice. **Fig. S5** p16 induction inhibits intestinal cell proliferation. **Fig. S6** No evidence for increased apoptosis in intestine with p16 induction. **Fig. S7** Crypt cell types that express p16 following sustained induction. **Fig. S8** Sustained p16 induction does not increase SAß-gal staining in intestine. **Fig. S9** p16 induction d20-40 in CMV-rtTA-TetO-p16 mice imposes aging features. **Fig. S10** p16 induction in skin is associated with fewer hair-filled follicles and thinner dermal fat layer. **Fig. S11** Regrowth of hair following Dox withdrawal in shaved CMV-rtTA:TetO-p16 mice. **Fig. S12** p16 induction in M2-rtTA:TetO-p16 mice causes premature aging features. **Fig. S13** p16 induction in intestine of CAG-rtTA3 mice usually results in uniform expression in the intestinal epithelium and is countered by increased cyclin D and Cdk4 expression (a-c) CAG-rtTA3:TetO-p16 mice were treated with Dox for 1 week near 1 month of age. Jejunal tissue was fixed in formalin and subjected to IHC for exogenous p16 (brown). **Fig. S14** Widespread p16 induction in CAG-rtTA3:TetO-p16 mice. **Fig. S15** Inhibition of BrdU incorporation and imposition of aging features in CAG-rtTA3:TetO-p16 mice and their rescue by germline Cdk4 R24C alleles. **Fig. S16** Marked hair loss in a CAG-rtTA3:TetO-p16 mouse treated with Dox despite normal weight. **Fig. S17** Induction of cyclin D and Cdk4 but not senescence-associated mRNAs in intestinal epithelial cells of a CAG-rtTA3:TetO-p16 mouse.

**Table S1** RT-PCR for senescecne markers Dec1 & DcR2. **Table S2** Sample Information.
