# Supplementary material for: Reversible cell cycle inhibition and premature aging features imposed by conditional expression of p16Ink4a
Source: Aging Cell. 2014 Dec 6;14(1):139–47. doi: 10.1111/acel.12279 (PMC4326901; doi:10.1111/acel.12279)
Supplement: Supplementary file 1 [file acel0014-0139-sd1.pdf]

Figure-S1 Enders

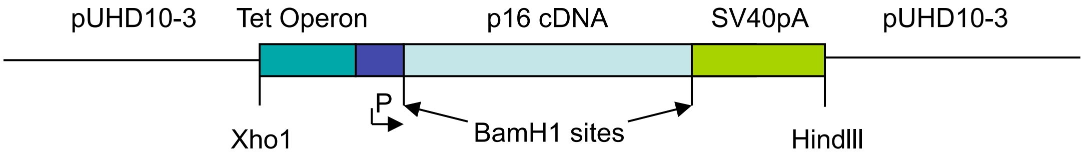

Figure-S2 Enders

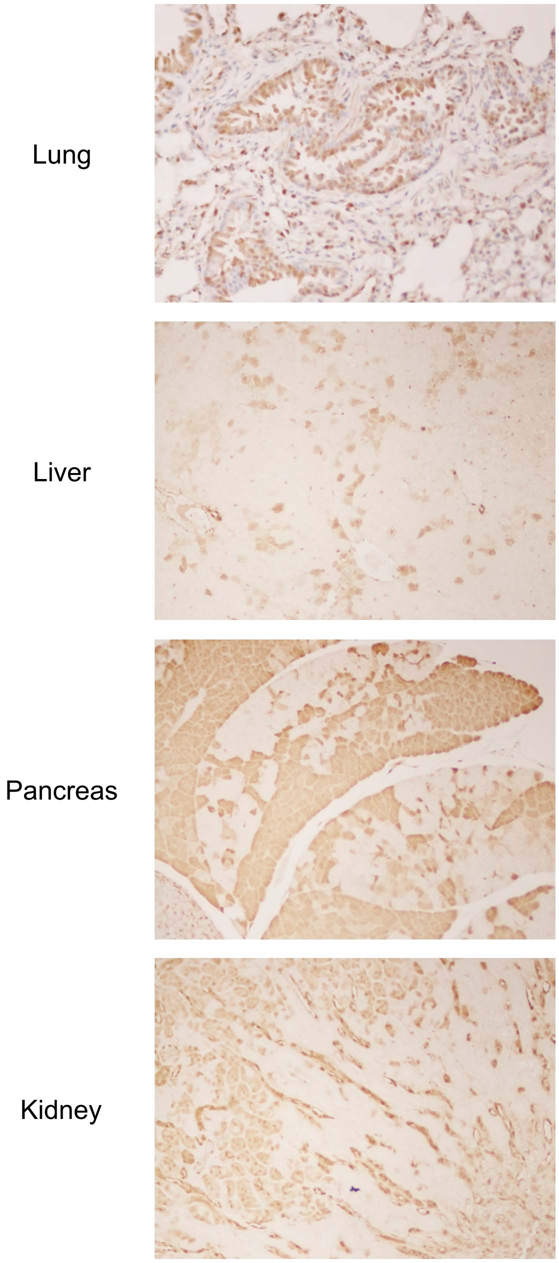

Figure-S3 Enders

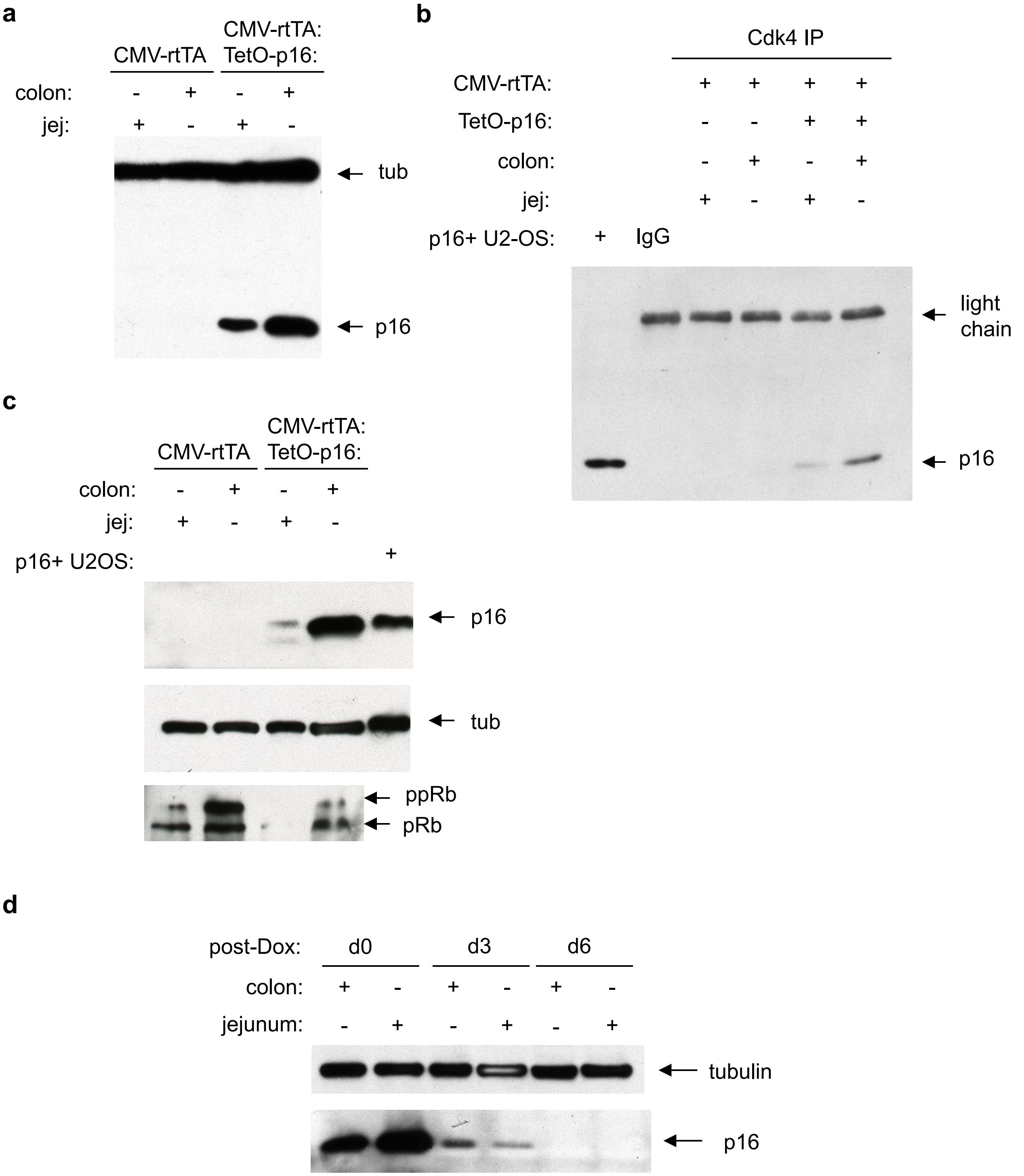

Figure-S4 Enders

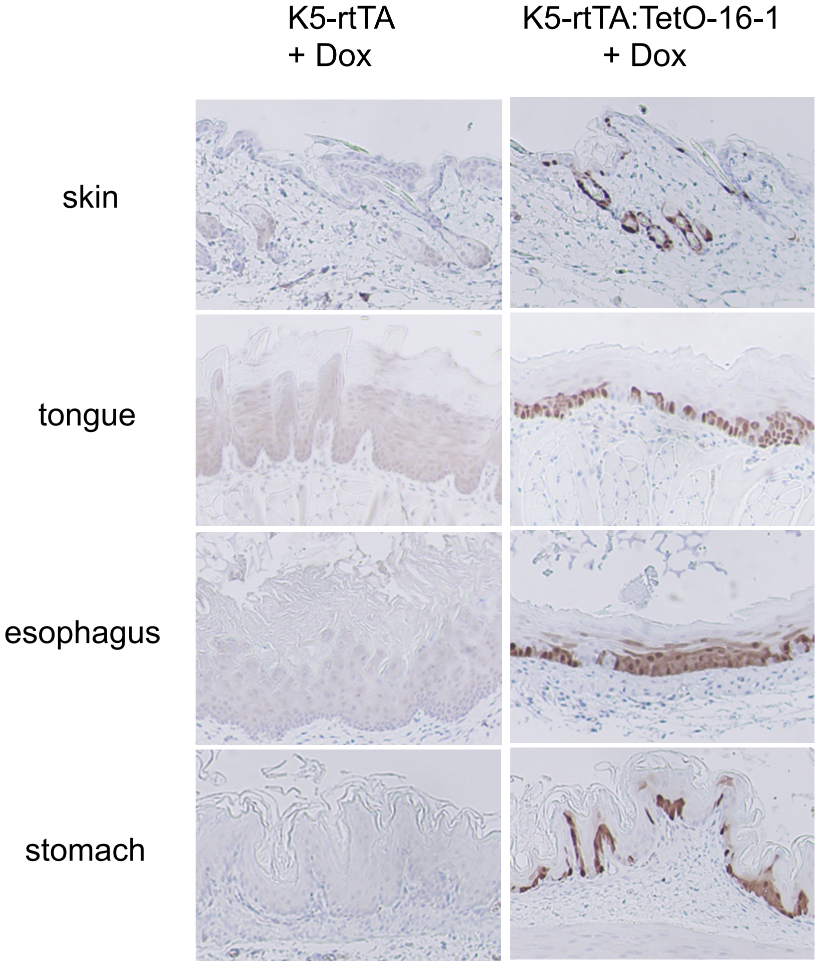

Figure-S5 Enders

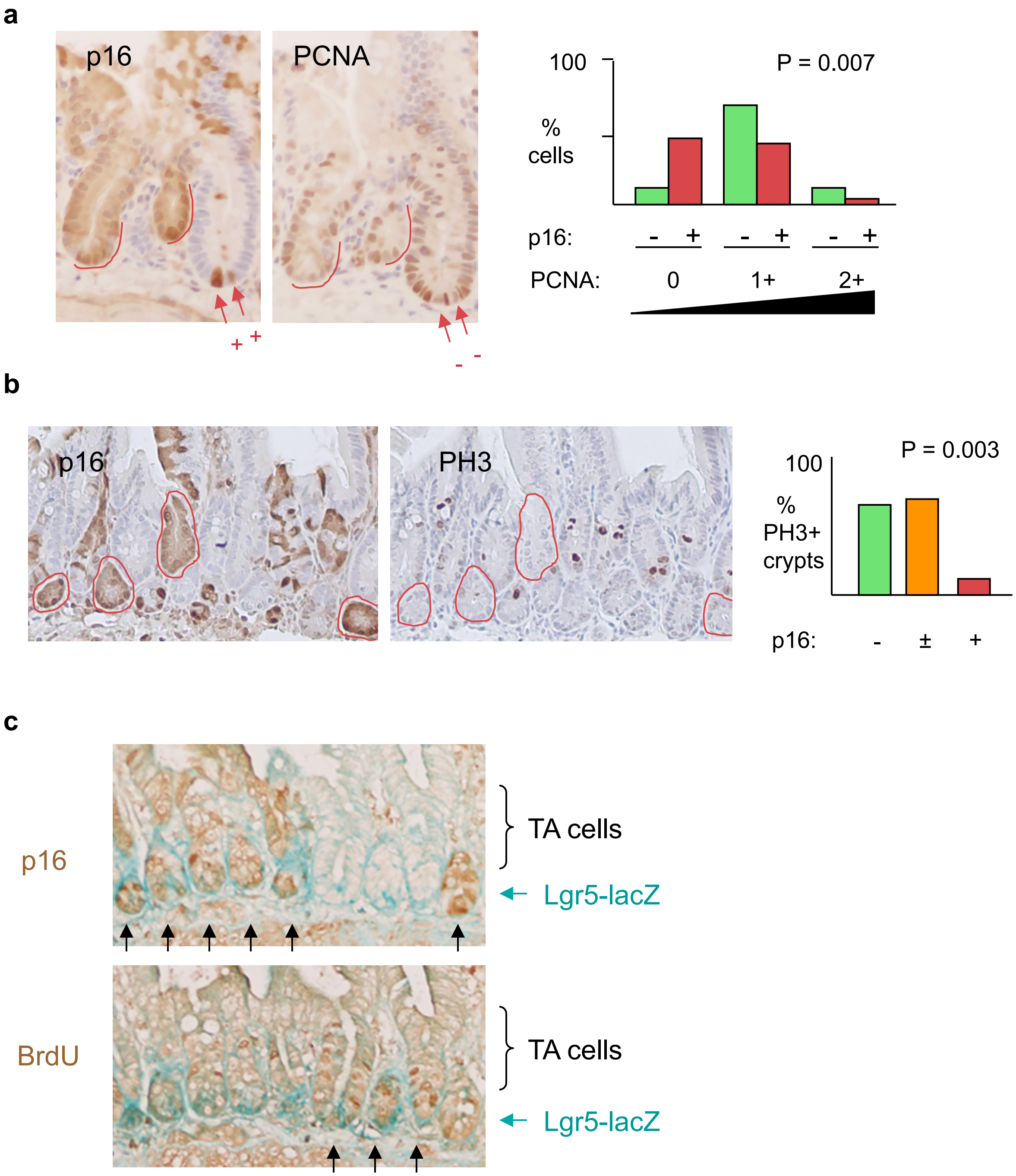

Figure-S6 Enders

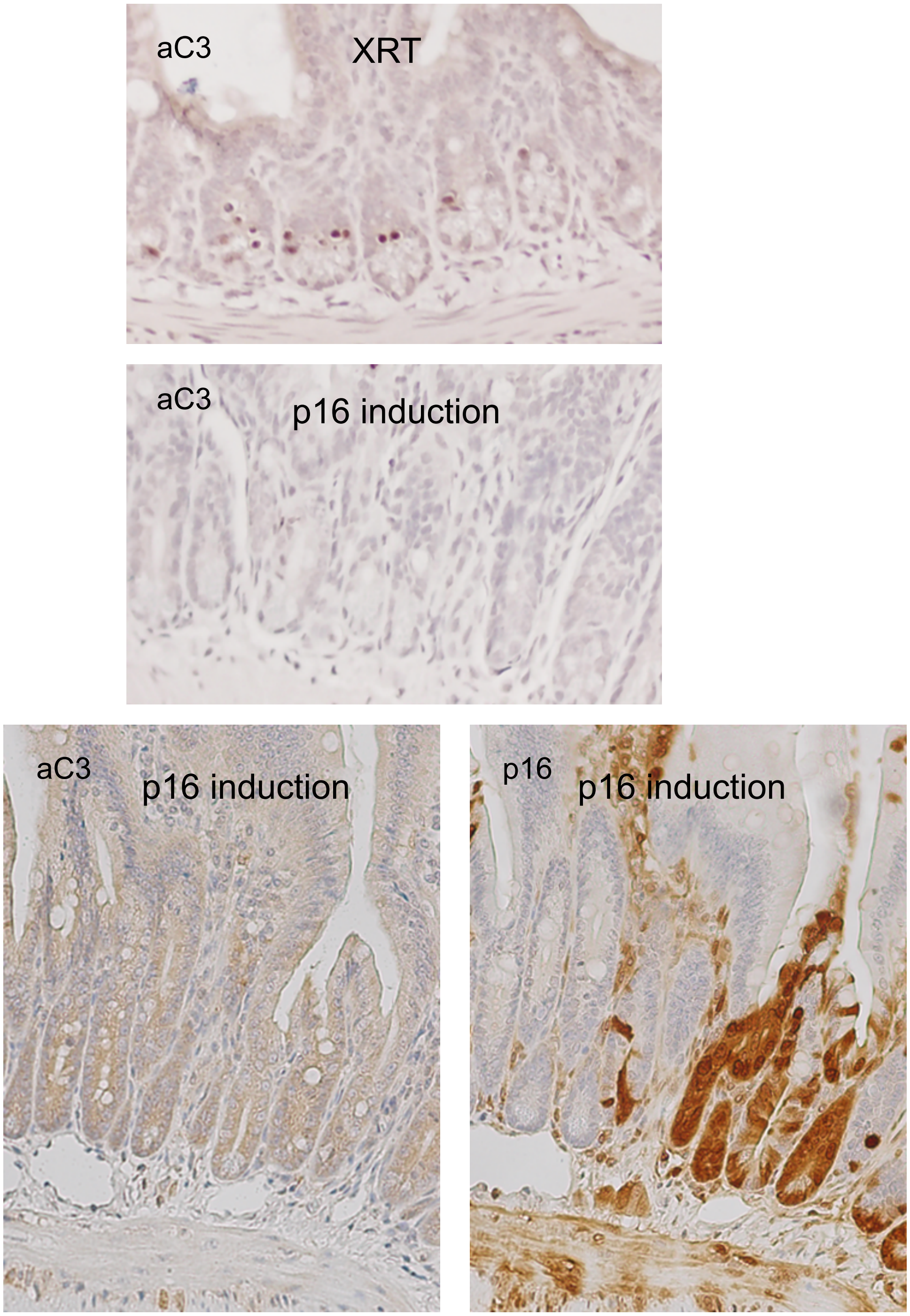


Serial sections:

Figure-S7 Enders

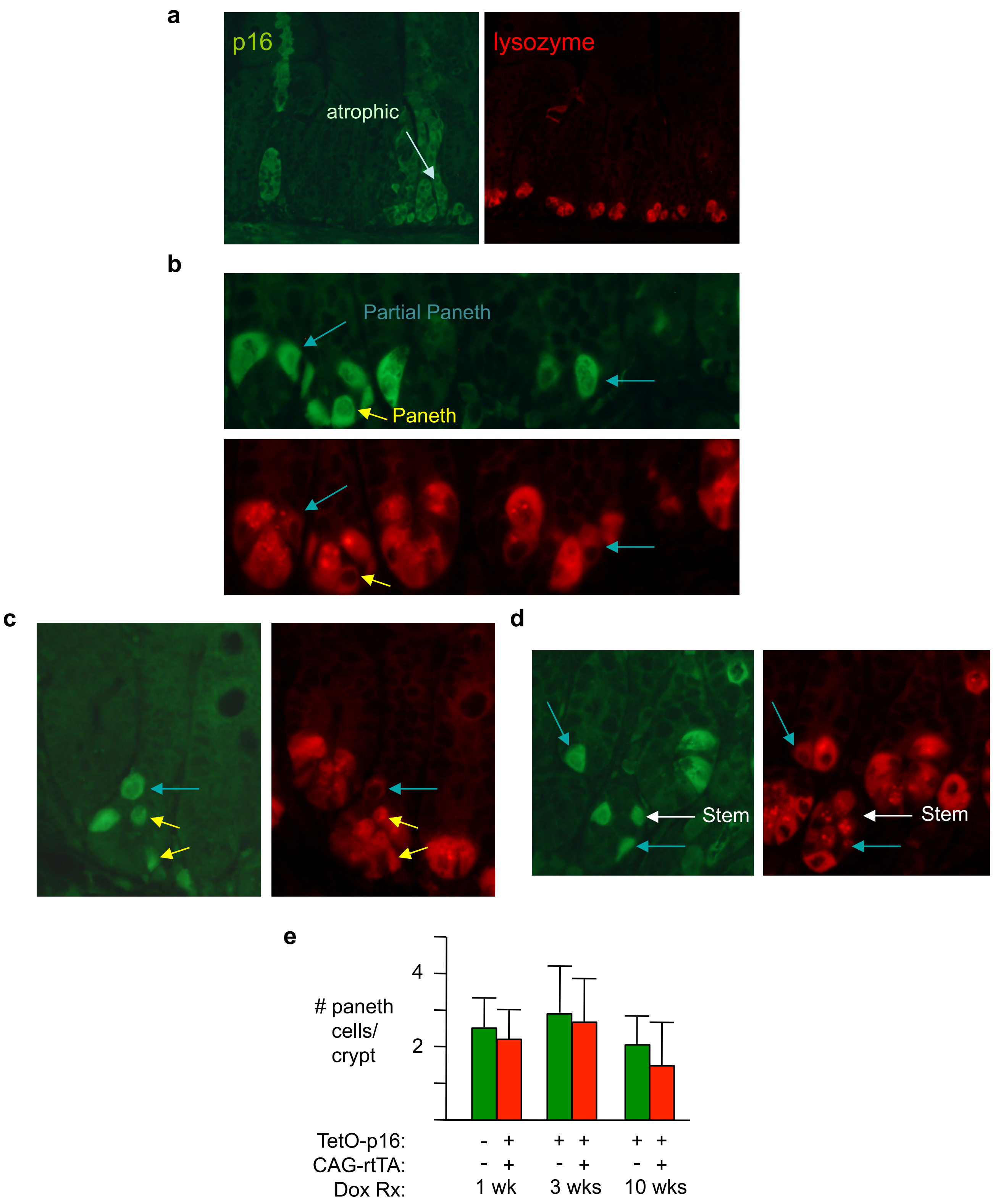

# Figure-S8 Enders

CAGrtTA3:
TetOp16

DBZ Rx

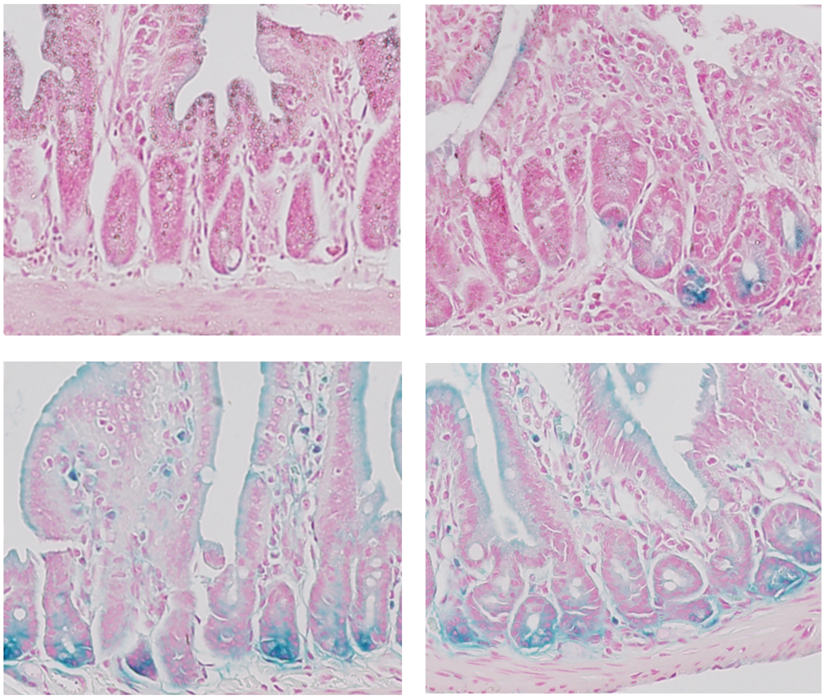

# Figure-S9 Enders

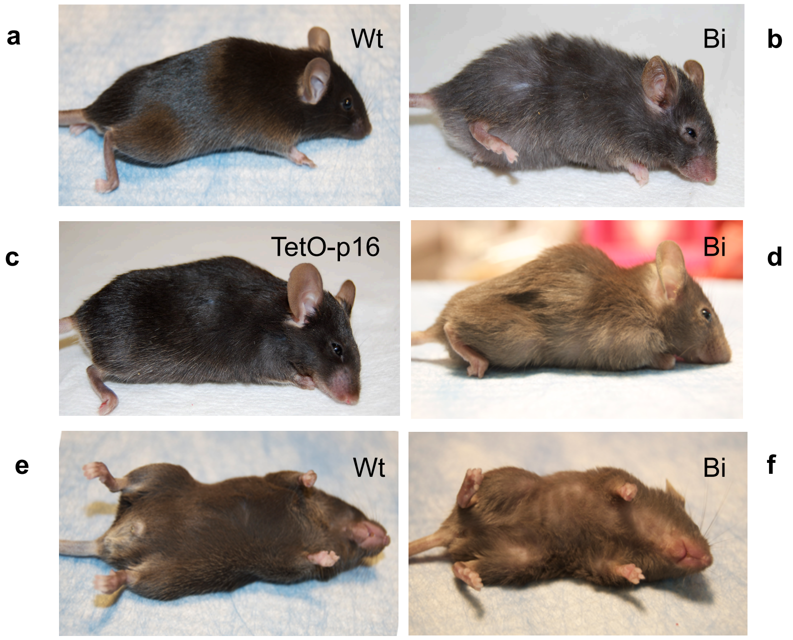

Figure-S10 Enders

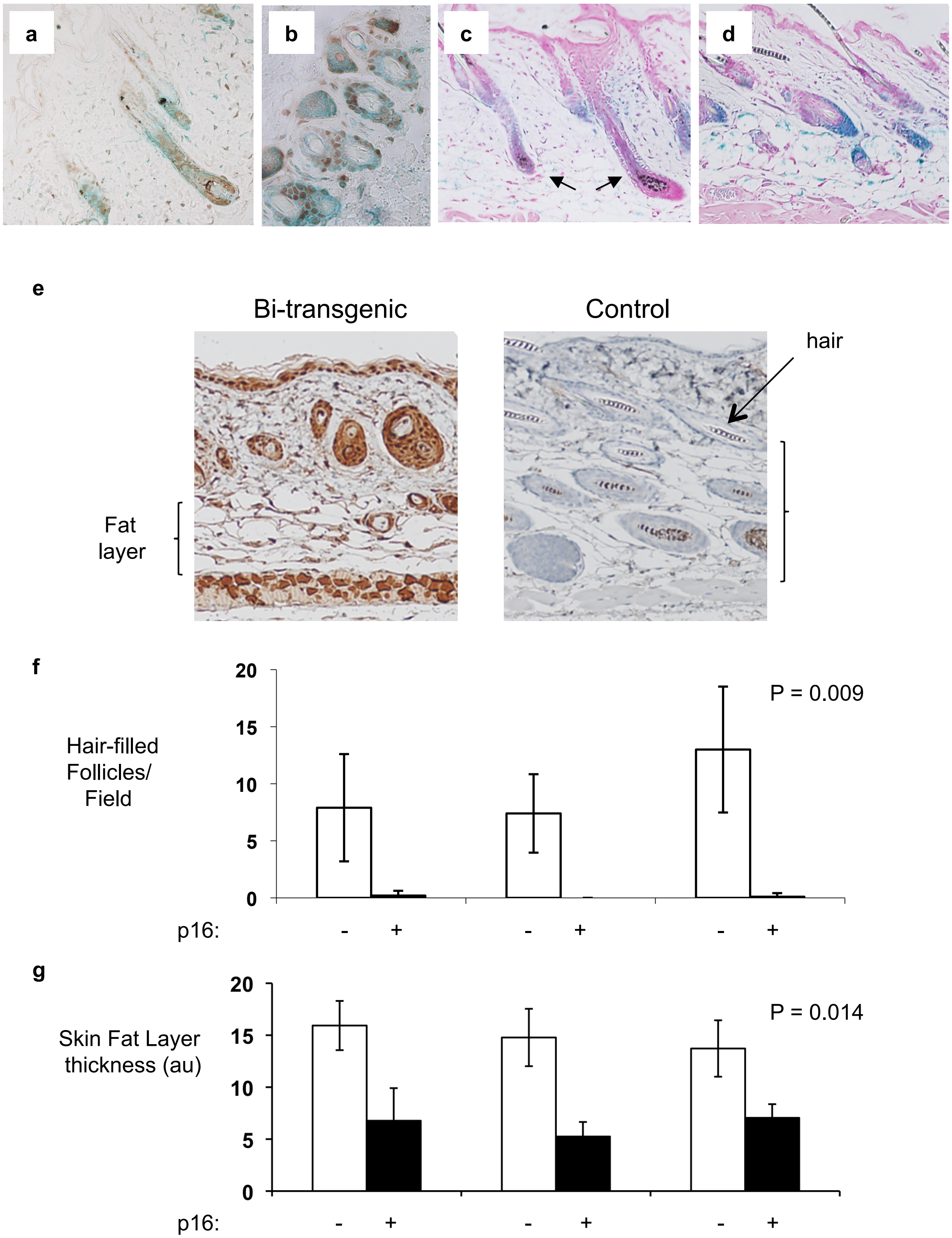

# Figure-S11 Enders

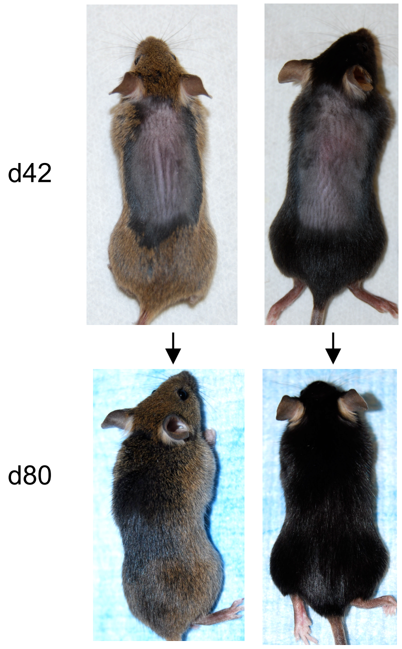

Figure-S12 Enders

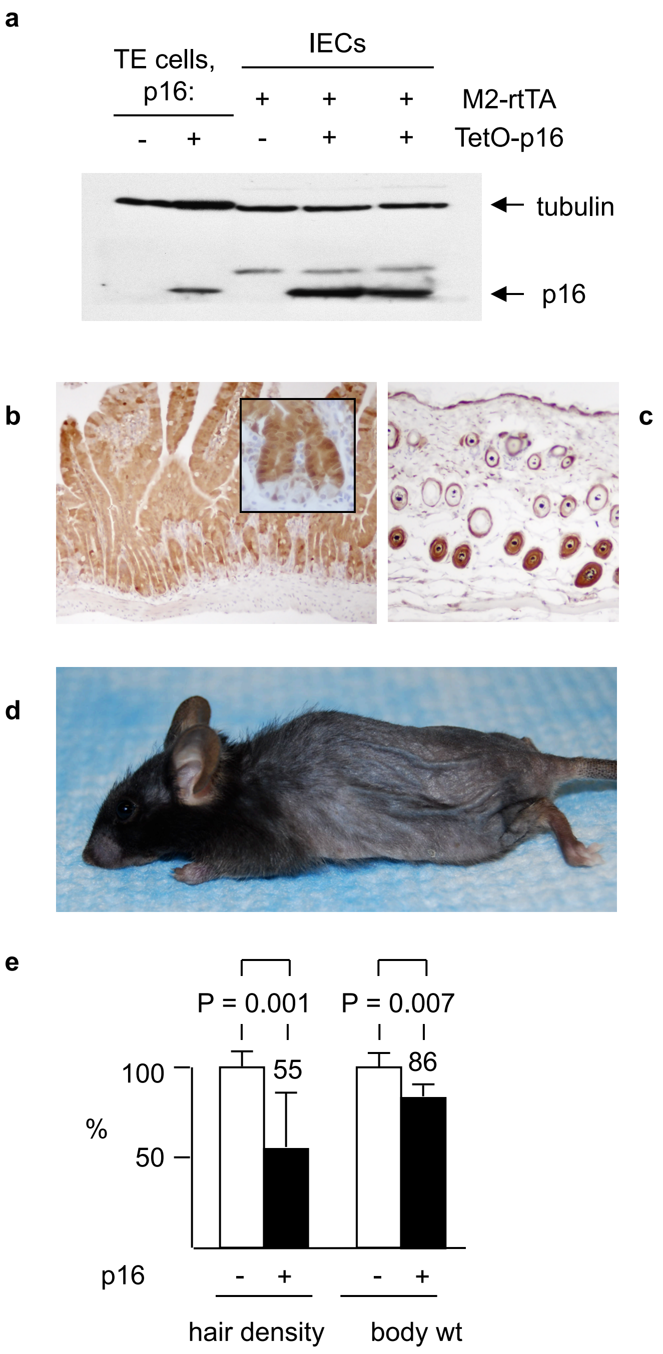

Figure-S13 Enders

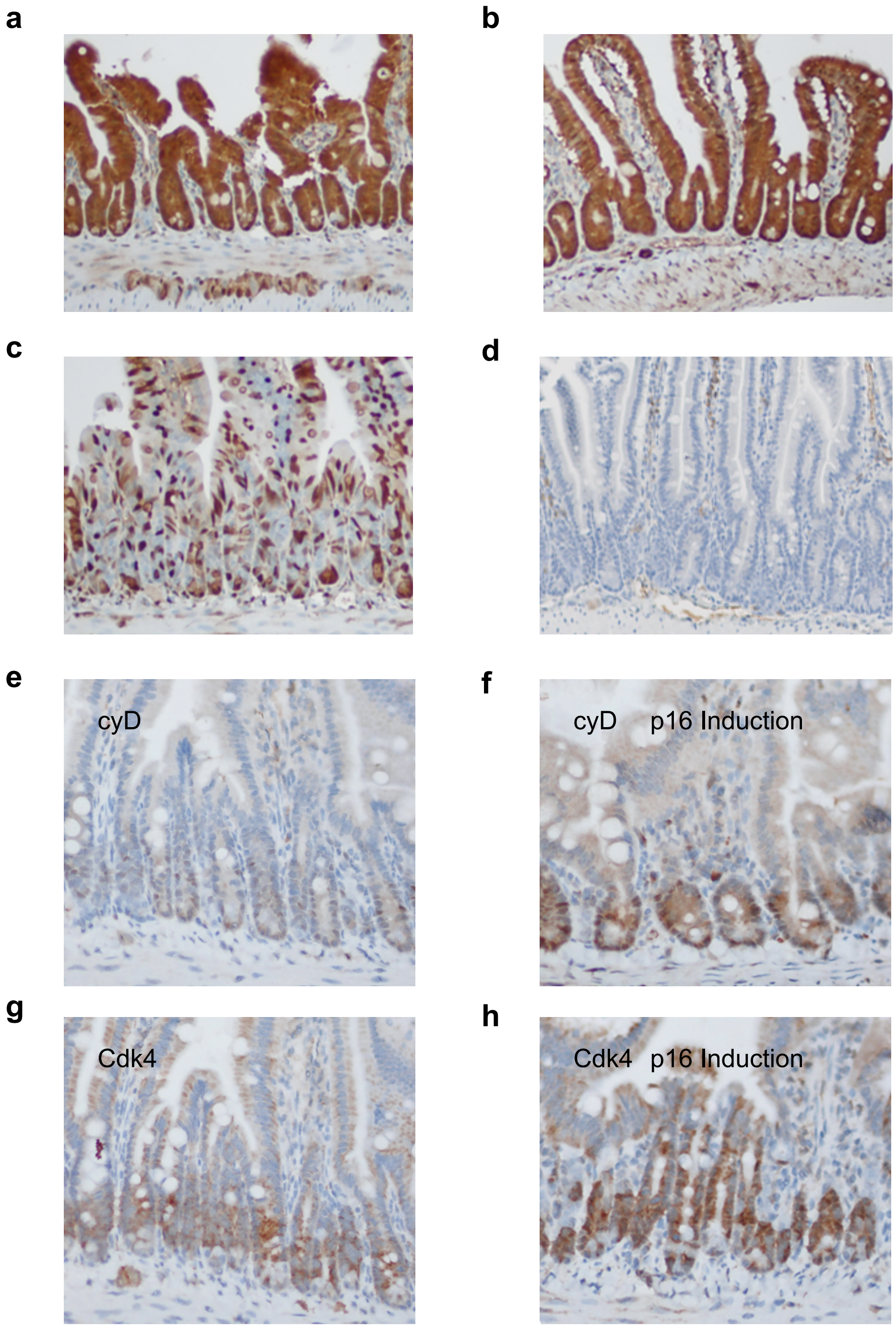

# Figure-S14 Enders

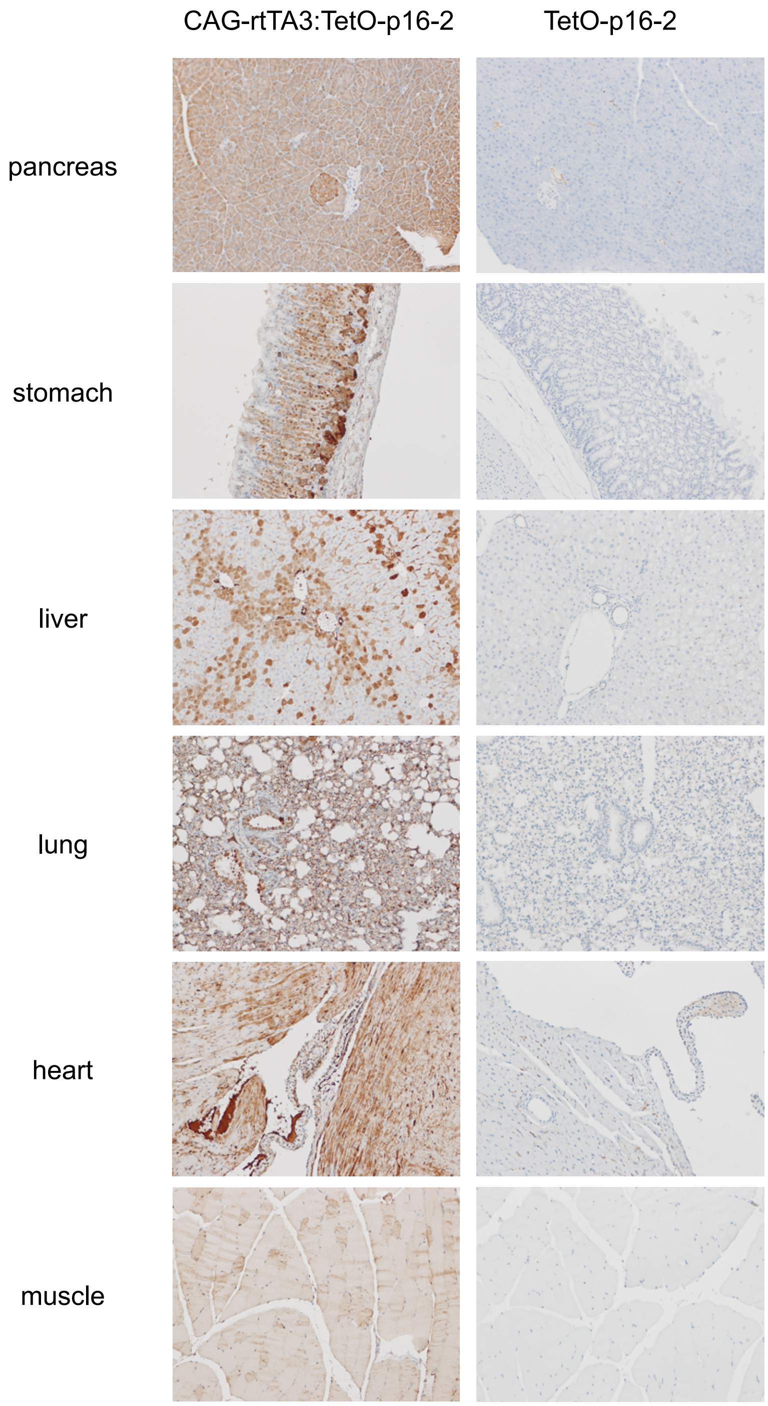

# Figure-S15 Enders

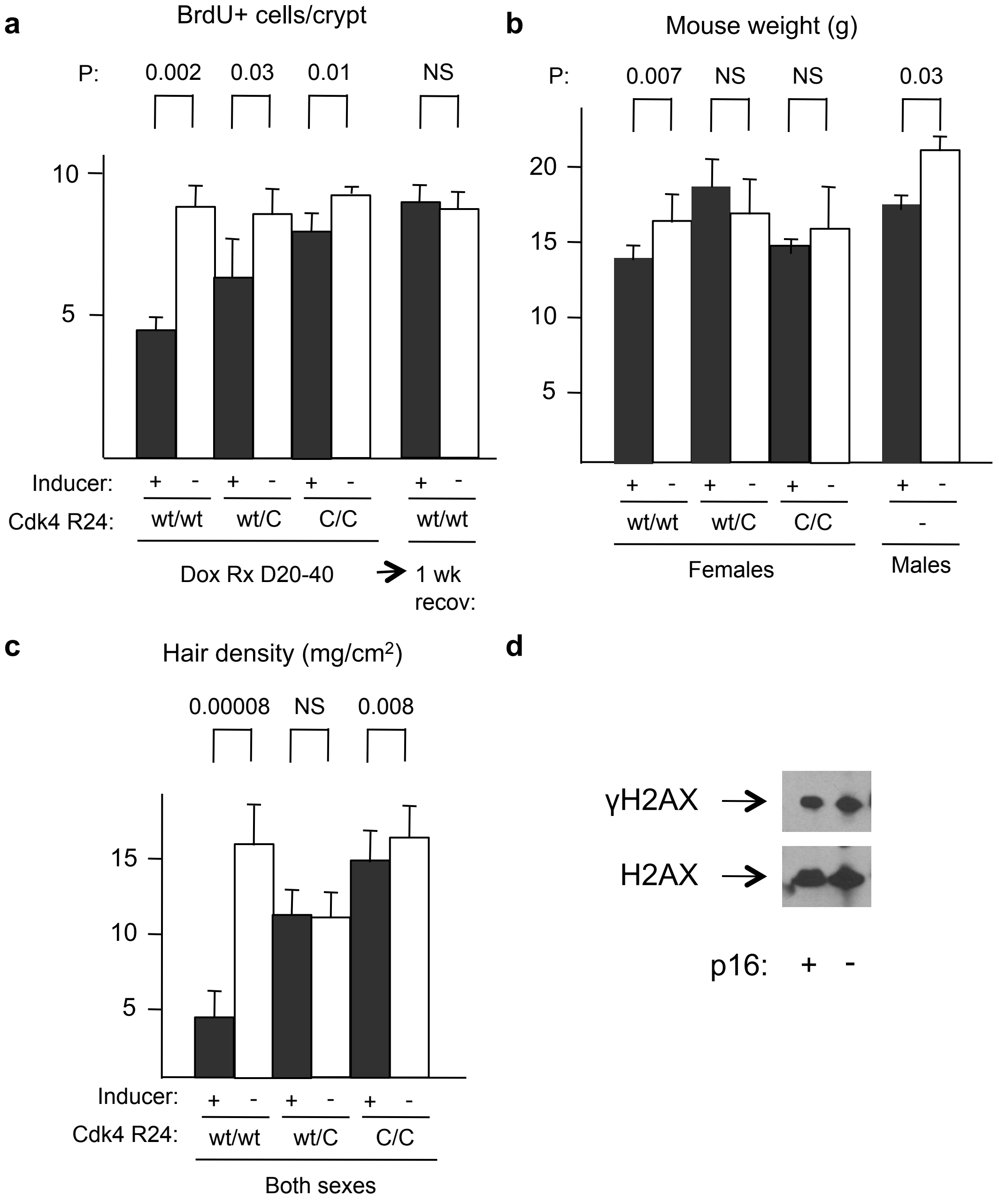

Figure-S16 Enders

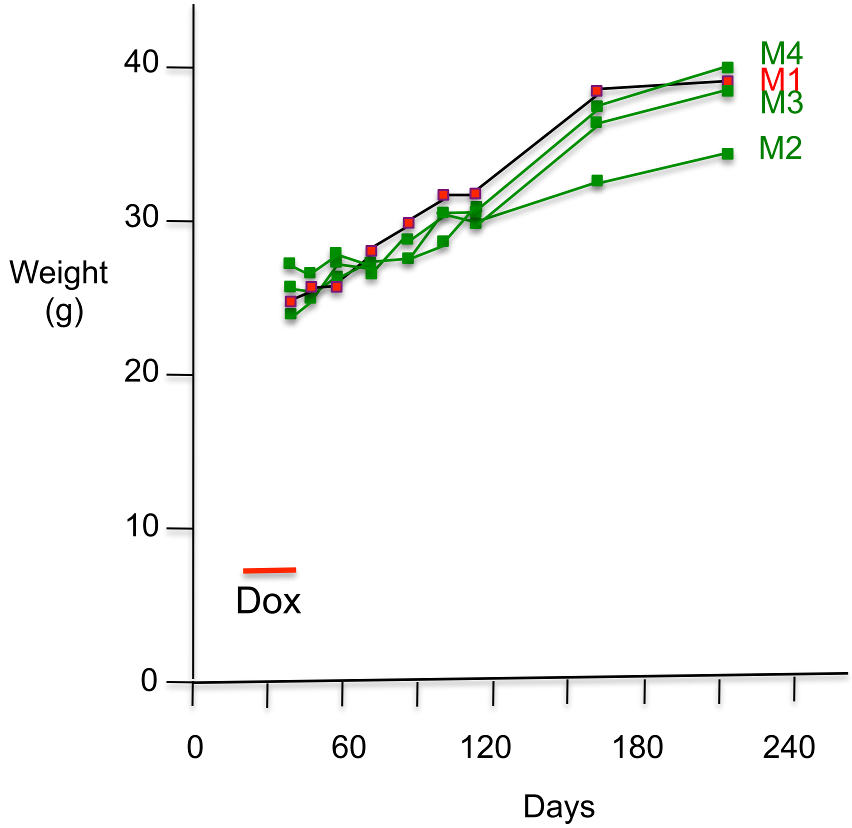


| Dox, Day | M4 | M1 |
| --- | --- | --- |
| Dox, 40 | | |
| Recovery, 70 | | |

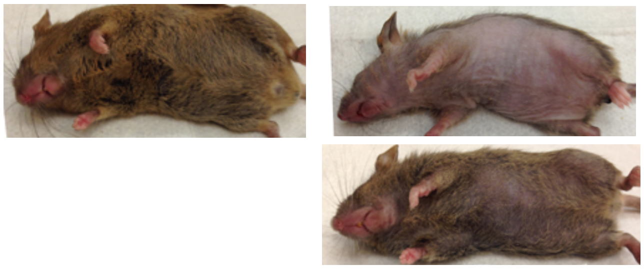

Figure S17-Enders

Dox Rx: D20-40

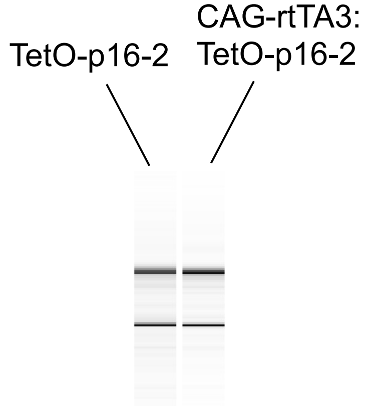


| **Gene** | **TetO-p16** | **CAG-rtTA3:TetO-p16** |
|---|---|---|
| CCND2 | 9.65 | 11.55 |
| Cdk4 | 7.6 | 8.6 |
| GM-CSF | 5.31 | 5.35 |
| Gro-a/CXCl1 | 4.8 | 4.98 |
| Gro-b/CXCL-2 | 5.44 | 5.2 |
| Gro-c/CXCL-3 | 4.08 | 2.88 |
| ICAM1 | 6.22 | 5.88 |
| HCP-1/CCL-2 | 4.82 | 4.79 |
| HCP-2/CCL-8 | 4 | 3.98 |
| HCP-3/CCL-7 | 5.89 | 5.5 |
| HCP-4 | 6.86 | 6.77 |
| DcR2/Tnfrsf10b | 7.88 | 7.96 |
| PAI-1/SERPINE-1 | 5.59 | 5.26 |
| IL-8 | 4.61 | 4.45 |
| IL-6 | 2.62 | 2.92 |
| OPG | 5.58 | 5.77 |

Log2 scale
